# Splenectomy in Banti's Disease

**Published:** 1913-12

**Authors:** E. Hey Groves

**Affiliations:** Surgeon to the Bristol General Hospital.


### SPLENECTOMY IN BANTI'S DISEASE.

BY

E. Hey Groves, M.S. Lond., F.R.C.S.,
*Surgeon to the Bristol General Hospital.*

It is now very widely accepted that removal of the spleen is called for in the majority of cases of splenomegaly, except those associated with leukæmia.

The spleen is probably more than a merely passive blood filter, and its hypertrophy denotes some metabolic error, even as does the enlargement of the thyroid gland.

In Banti's disease there is an anæmia, followed or accompanied by splenic enlargement, and then at a later date occurs cirrhosis of the liver, ascites, jaundice and death. Now whether the *causa causans* of this disease is in the spleen or in the blood, there is sufficient reason to believe that the spleen is the essential factor in its development, and therefore it ought to be removed as soon as the nature of the disease is recognised.

There can be no doubt that the splenectomy ought to take place before any profound change has occurred in the liver, because nothing can restore hepatic function after a certain stage of cirrhosis has occurred. But that even in this stage, removal of the spleen may sometimes be undertaken with benefit, is shown by the following case.

X. Y., a married lady, without family aged 40 years, was born in India, and is said to have had a large spleen since early infancy. More than a year prior to the date of operation, she began to suffer with abdominal discomfort, attacks of bilious vomiting, and general malaise. She consulted Sir Patrick Manson, who detected some enlargement of the liver as well as that of the spleen, but did not suggest any active treatment. For some months the abdomen has been rapidly enlarging.

*Condition prior of operation.*—There was very great abdominal distension, which was obviously caused by free fluid. The spleen descended to the umbilicus. The liver was not felt. There was some œdema of the feet. Other organs and the urine were normal.

The blood was practically normal, red cells 6,000,000, white 16,000, with 71 per cent. polymorphs, hæmoglobin rather over normal.

*Operation, August 20th,* 1913.—Abdomen opened by a medium incision above the umbilicus, allowing the escape of many pints of a straw-coloured fluid. The liver was grossly deformed, being shrunken and contracted into two round irregular masses, representing the right and left lobes, neither of which came forward as far as the costal margin.

The incision was enlarged by a transverse cut through the left rectus, in a line between the navel and ensiform cartilage. The spleen was removed (weighing $3\frac{1}{2}$ lb.) after ligature and division of its very vascular pedicle. The main artery and vein seemed to be about the thickness of one's little finger. The operation was completed by fixing the omentum into the deep layers of the abdominal wound, so as to relieve the hepatic circulation.

*The after history* for two months was not very encouraging, for although the patient all the time expressed herself as feeling much better, and especially in respect to the absence of the bilious vomiting, yet the accumulation of ascitic fluid was extraordinarily rapid. Every six or seven days she had to be tapped, and often as much as ten pints of fluid withdrawn. Directly the abdomen became tense the legs showed marked œdema. Towards the end of the second month, however, the ascites became less rapid in its development, and at the present time I am informed by her medical man that she only requires tapping every three weeks, and that this only produces a few pints of fluid. She is now resuming an active life, and is in every way unquestionably the better for the operation.

In considering the condition which exists in such a case as this, there is ample reason to expect the removal of the spleen to be a relief in even advanced cirrhosis. In the normal anatomical condition, the splenic vein forms about one-third of the total portal tributaries, and in such a case as the above it probably would convey more blood to the portal vein than all the other feeders of this vessel put together. Therefore the mere removal of the spleen takes away half the blood going into the narrowed hepatic circulation, and allows the blood from the digestive tract to monopolise the liver functions.

Of course, the ultimate relief or cure of the ascites in such a case can only come about by means of the anastomotic circulation afforded by the omentopexy.

---

